# *SLC40A1*-related hemochromatosis associated with a p.Y333H mutation in mainland China: a pedigree report and literature review

**DOI:** 10.1186/s12920-024-01929-0

**Published:** 2024-06-17

**Authors:** Yue Li, Fangfang Duan, Song Yang

**Affiliations:** 1 Division 3, Department of Hepatology, Beijing Ditan Hospital, Capital Medical University, Beijing, 100015 China; 2 Division 2, Department of Hepatology, The Fourth People’s Hospital of Qinghai Province, Qinghai, 810000 China; 3 Center of Hepatology, Beijing Ditan Hospital, Capital Medical University, 8 Jingshun East Street, Beijing, 100015 China

**Keywords:** Hemochromatosis, SLC40A1, Ferroportin, Phenotype, Pedigree

## Abstract

**Background:**

Haemochromatosis is a genetic disease characterized by the excessive deposition of iron in various tissues and organs, eventually results in organ damage including cirrhosis, diabetes, cardiomyopathy, etc. *SLC40A1*-related haemochromatosis is associated with gain-of-function mutations in the *SLC40A1* gene, which encodes ferroportin. While sporadic reports of this condition exist in mainland China, the understanding of the phenotype and genetic pattern associated with the *SLC40A1* p.Y333H mutation remains incomplete.

**Case presentation:**

We report a pedigree with heterozygous p.Y333H mutation in Chinese Han population. The proband is a 64-year-old man complaining of persistent abnormality of liver enzyme levels for 1 year, with a history of knee joint pain, diabetes and skin pigmentation. He displayed markedly elevated serum ferritin level and transferrin saturation. Magnetic resonance imaging showed iron deposition in the liver, spleen, and pancreas, along with cirrhosis and splenomegaly. Whole exome sequencing identified a heterozygous allelic variant c.997T > C (p.Y333H). Genetic screening of family members identified four first-degree relatives and three second-degree relatives having the same mutation. Additional cases with this mutation from two published studies were included. Among the probands and screened relatives, all eight males aged over 30 y had ferritin level > 1000 µg/L, transferrin saturation > 90%. Four patients with organ damage in the present study received therapeutic phlebotomy, alleviating clinical symptoms and improving in transferrin saturation and serum ferritin.

**Conclusions:**

This study reports the largest pedigree with heterozygous *SLC40A1* p.Y333H mutation in the Chinese population to date. In Chinese families, males over 30 years old with hemochromatosis due to *SLC40A1* p.Y333H mutation exhibit severe iron overload phenotypes.

## Background

Haemochromatosis (HC) is a genetic disease characterized by the excessive deposition of iron in various tissues and organs, eventually results in organ damage including liver fibrosis/cirrhosis, diabetes, arthropathy, cardiomyopathy and hypogonadism [1, 2]. As a rare autosomal dominant inherited disease, *SLC40A1*-related HC is caused by gain-of-function mutation in the *SLC40A1* gene encoding ferroportin (FPN) [3, 4]. The mutated FPN exhibits resistance to hepcidin’s inhibitory effects, causing an excessive iron efflux to plasma and subsequently iron deposition in parenchymal cells [5]. The potential pathogenesis involves FPN variants impairing the hepcidin binding, or altering hepcidin-induced conformational change in FPN, leading to impaired FPN ubiquitination [6].

The pathogenic genes of HC in the Asian population are mainly non-*HFE* genes, which are different from those in the European population [7]. A recent study in the Chinese population showed that, among 32 patients with primary iron overload, 31 harbored non-*HFE* variants, with *HJV* (8/31) and *SCL40A1* (7/31) variants being particularly prominent [8]. This underscores *SLC40A1*-related HC as a major etiological factor in HC cases in China. Gain-of-function mutations in the *SLC40A1* gene are often private and sporadically reported in China, including missense mutations like p.N144D, p.C326F, p.C326Y, p.Y333H and p.V511I, as well as splicing mutations IVS3 + 10 del GTT and IVS1-8 C/G [8–13].

Previous studies have highlighted the p.Y333H mutation as a genetic variation in the Chinese population [8, 12, 13]. A comprehensive understanding of the phenotype and genetic pattern of HC associated with this genotype is lacking. In this study, we report a pedigree with a heterozygous p.Y333H mutation in the Chinese Han population, analyze the clinical characteristics and describe the penetrance and expression of iron overload in *SLC40A1*-related HC with this mutation.

## Case presentation

**Fig. 1:**
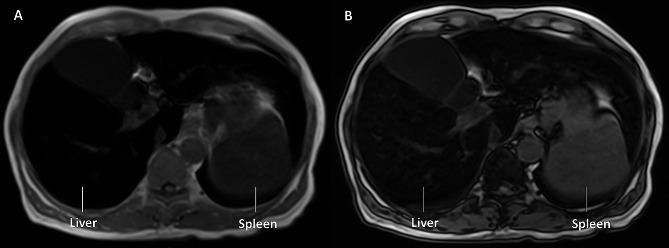
Dual-sequence magnetic resonance imaging (MRI) of the proband with *SLC40A1*-related haemochromatosis. The T1-weighted imaging displayed hypo-signal intensity in the liver and spleen, and the signal intensity of liver was lower than that of spleen. The signal intensity of the liver at axial gradient-echo (GRE) T1-weighted in-phase sequence (**A**) was lower than that at the out-of-phase sequence (**B**)

The proband (H1), a 64-year-old male, initially visited our hospital with a one-year history of persistent abnormal liver enzyme levels. He presented intermittent mild abdominal distention, dyspepsia, and sustained mild elevation of aminotransferase levels in the recent year. The patient had a history of knee joint pain, a diagnosis of diabetes mellitus with insulin treatment, and no reported history of alcoholism, blood transfusion, or iron supplementation. Physical examination revealed skin pigmentation and laboratory results indicated a slight increase in aminotransferase and gamma-glutamyl transferase, along with decreased leukocyte and platelet count (associated with hypersplenism), hyperglycemia and hyperlipidemia. The proband displayed normal in terms of hepatitis virus markers, inflammatory parameters, tumor markers, ceruloplasmin. Iron store indices revealed markedly elevated serum ferritin (SF) level (5061 µg/L) and transferrin saturation (TS) (99.1%). Abdominal magnetic resonance imaging (MRI) of the proband (Fig. 1) displayed T1-weighted and T2-weighted hypo-signal in the liver, spleen, pancreas and vertebrae, indicating tissue and organ iron overload. MRI also identified cirrhosis, hepatomegaly, splenomegaly, esophageal and gastric varices, and knee joint osteochondral injury. Dual-energy x-ray absorptiometry exhibited osteopenia.

**Fig. 2:**
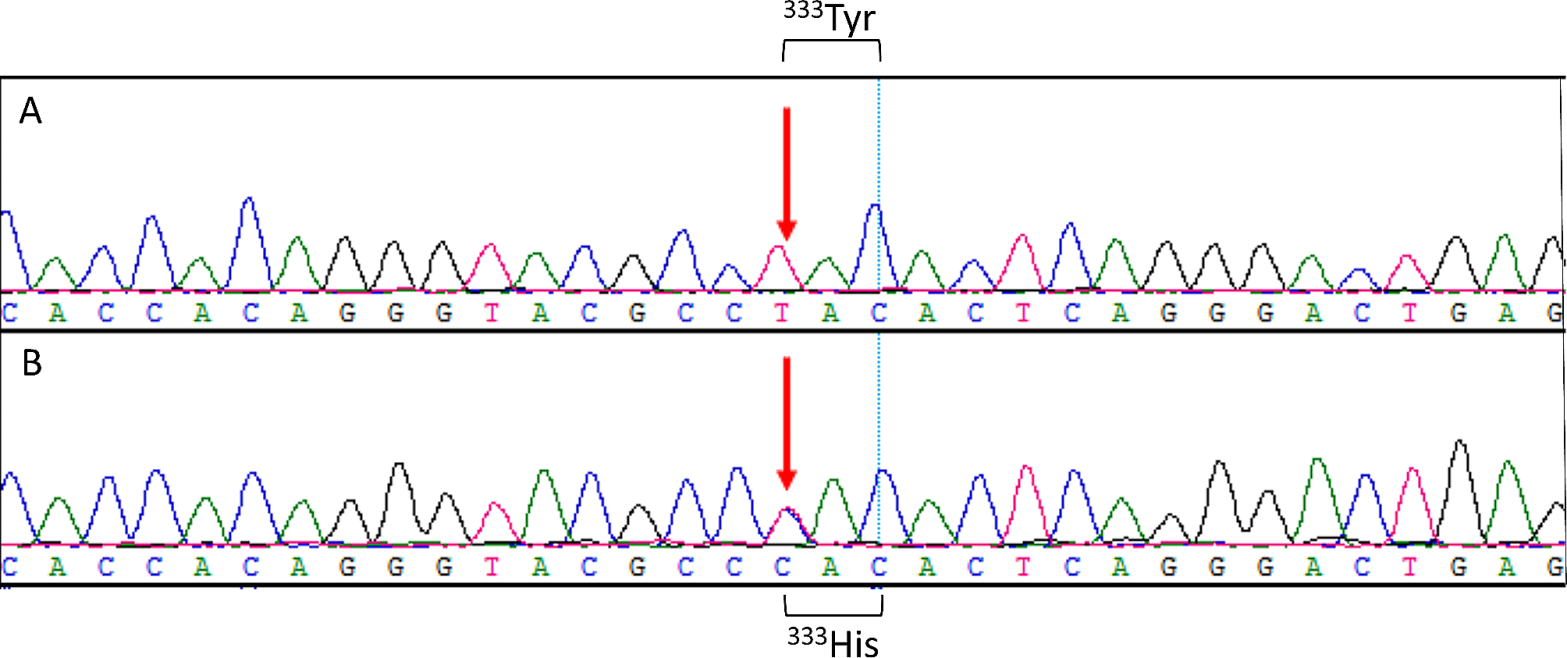
Genetic testing of the proband with the heterozygous p.Y333H mutation in *SLC40A1* gene. Sequence analysis of the *SLC40A1* gene from a control* (**A**) and the proband (**B**) indicated a heterozygous missense mutation, c.997T > C (red arrow) in the exon 7 of *SLC40A1*, which resulted in the substitution of tyrosine at position 333 by histidine. * The control was a sister of the proband without *SCL40A1* mutation

Whole exome sequencing identified a heterozygous allelic variant c.997T > C in exon 7 of *SLC40A1* (Fig. 2), resulting in a tyrosine-to-histidine substitution at amino acid position 333 (p.Y333H). It has been proven to be a gain-of-function mutation, representing a dominantly inherited *SLC40A1*-related HC [12]. No mutations or variants were identified in other HC-related genes (*HFE*, *HAMP*, *HJV*, *TFR2, BMP6*). According to the diagnostic criteria of relevant guideline [2], the proband was diagnosed as *SLC40A1*-related HC, displaying cirrhosis, diabetes and arthropathy.

**Fig. 3:**
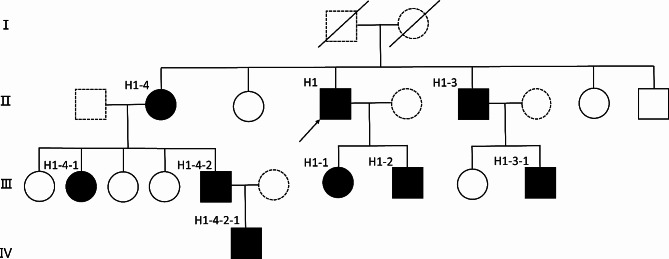
Segregation analysis of *SLC40A1*-related hemochromatosis with the p.Y333H mutation is consistent with autosomal dominant inheritance. Black square/circle indicates male or female with *SLC40A1*-related hemochromatosis, who had heterozygous p.Y333H mutation. White square/circle indicates male or female without the p.Y333H mutation. Dashed square/circle represents male or female didn’t perform the genetic testing. The arrow indicates the proband

Genetic testing of seven existing first-degree relatives revealed that the proband’s daughter (H1-1), son (H1-2) and two siblings (H1-3, H1-4) had p.Y333H mutation in a heterozygous state. Further genetic testing of eight next-degree relatives of the two siblings (H1-3, H1-4) identified four individuals with p.Y333H heterozygosity. The pedigree analysis is depicted in Fig. 3.

Owing to the practical limitations, medical history collection, serum iron indices and abdominal MRI were available for four additional heterozygotes within the family. Table 1 summarizes the clinical manifestations of the proband and his heterozygous family members.

**Table 1:** Clinical characteristics of Chinese heterozygotes with p.Y333H mutation in *SLC40A1* gene

Patient number	Sex	Age	Iron test		Liver enzyme levels		Iron deposition on MRI	Organ damage manifestations
			SF (µg/L)	TS (%)	ALT (U/L)	AST (U/L)		
H1 (proband)	M	64	5061	99.1	90.5	72.1	Liver, spleen, pancreas, vertebrae	Cirrhosis, diabetes, arthropathy, osteopenia
H1-1 (daughter)	F	32	20.9	23.3	11.2	22	No deposition	No damage
H1-2 (son)	M	30	1263.7	97.6	58.1	34.8	Liver	Abnormal liver enzymes
H1-3 (brother)	M	59	> 1500	95.6	81.8	38.4	Liver	Cirrhosis, arthropathy
H1-3-1 (nephew)	M	30	> 1500	96.5	93.2	33.5	Liver	Liver fibrosis, diabetes
H2^a^ (proband)	M	49	7445	97.3	83	87	Liver, spleen, pancreas	Cirrhosis, diabetes, arthropathy, loss of libido
H2-1^a^ (son)	M	16	120	69.6	24	19	NA	No damage
H3^a^ (proband)	M	60	15000	94.0	304	198	Liver, spleen, pancreas	Cirrhosis, diabetes
H4^a^ (proband)	F	66	1446	92.7	17	33	Liver, spleen, pancreas	Cirrhosis, diabetes
H4-1^a^ (son)	M	38	2332	92.0	73	32.5	Liver	Abnormal liver enzymes
H5^b^ (proband)	F	53	5651.3	99.4	200	84	Liver	Abnormal liver enzymes
H5-1^b^ (daughter)	F	30	190.1	80.0	12.3	16.2	NA	No damage

^a^ The patients are sourced from study by Zhang et al. [12]

^b^ The patients are sourced from study by Hu et al. [13]

Abbreviations: SF, serum ferritin (normal range: 23.9–336.2 µg/L); TS, transferrin saturation (normal range: 20-50%); ALT, alanine aminotransferase (normal range: 9–50 U/L); AST, aspartate aminotransferase (normal range: 15–40 U/L); MRI, abdominal magnetic resonance imaging; NA, not available

The proband’s 30-year-old son (H1-2), 59-year-old brother (H1-3), and 30-year-old brother’s son (H1-3-1) all showed mild elevation of aminotransferase, significant elevation of SF and TS, and iron deposition in the liver on MRI. H1-3 with a history of lower limb joint pain manifested as cirrhosis. H1-3-1 was obese and diagnosed as diabetes and liver fibrosis. In contrast, the 32-year-old daughter (H1-1) of the proband exhibited normal SF and TS, with no iron deposition on imaging, indicating only genetic susceptibility.

After being diagnosed with *SLC40A1*-related HC, the proband and his son received therapeutic phlebotomy, removing 400 mL of blood every 3–4 weeks. The procedure was well-tolerated. After nearly three years of treatment, SF level of the proband decreased by approximately 77% (1158 µg/L), and TS decreased to 69.7%. The proband’s discomfort symptoms and skin pigmentation were relieved, while joint pain remained. Although aminotransferase remained slightly elevated, decompensated cirrhosis or hepatocellular cancer did not develop. After receiving phlebotomy, the son’s SF level dropped below 100 µg/L, TS and liver enzymes normalized. The proband’s daughter maintained normal SF, TS and liver enzyme levels during nearly three years of follow-up. The patient H1-3 and his son H1-3-1 received regular therapeutic phlebotomy at a local hospital, experiencing varying degrees of symptom alleviation and improvement in SF and TS.

## Discussion and conclusions

In the present study, we report a pedigree with heterozygous p.Y333H mutation of *SLC40A1* gene in the Chinese population. The pedigree chart illustrates the mutation transmission from generation to generation. Notably, in the proband’s generation and the subsequent generation, there was no significant gender difference in p.Y333H mutation heterozygotes (male/female: 5/3, *p* = 0.119), and the overall genetic mutation rate was nearly 50% (8/15). In summary, the p.Y333H mutation of *SLC40A1* gene is consistent with autosomal dominant inheritance.

Given the rarity of the *SLC40A1* p.Y333H mutation, only two teams have reported this mutation in the Chinese population to date. In order to summarize the phenotypic characteristics of *SLC40A1*-related HC with the mutation, we incorporated independent cases with this mutation from the studies of Zhang et al. and Hu et al. [12, 13], as showed in Table 1. Among the five probands, the male to female ratio was 4:1, and the onset age ranged from 49 to 66 years, all of whom had iron deposition to the degree that organ damage occurred. Cirrhosis (4/5) and diabetes (4/5) were more prevalent in these probands, and arthropathy occurred in 2 probands. These findings suggest that the phenotypic characteristics of the probands with p.Y333H mutation are similar to those of adult-form HC (*HFE*-related and *TFR2*-related HC), including the later onset age, higher penetrance in males, slower disease progression in females, and common organ damage such as cirrhosis and diabetes, while cardiopathy and hypogonadism as more common damage in juvenile-form HC (*HJV*-related and *HAMP*-related HC) [5, 14].

The risk of clinical penetrance of HC increases with age [15, 16]. In the overall population of probands and screened relatives with p.Y333H mutation, all eight males over 30 years old had progressed to liver iron deposition on MRI and varying degrees of organ damage in the liver and/or pancreas. Conversely, the 16-year-old male H2-1 presented with moderate increase in TS, normal SF, and no signs of organ iron deposition. This observation may indicate a relatively high clinical penetrance of the mutation in males over 30 years old. Compared to the late onset age in the five probands, three males (H1-2, H1-3-1, H4-1) from family screening had progressed to organ iron deposition in their thirties. These findings underscore the importance of genetic screening in first-degree relatives of p.Y3333H variants, especially males over 30 years old, as they may already be in the clinical onset stage. After identifying these individuals, it is crucial that timely and effective phlebotomy can significantly improve their prognosis [17].

Among young women, H1-1(32 y) and H5-1 (30 y) only had normal or moderately elevated TS without organ iron deposition. It implies that female patients with the mutation have later onset of penetrance and milder phenotypic expression, consistent with reported gender prevalence in adult-form HC [2, 5, 14]. The protective effect of premenopausal women on penetrance is mainly due to iron loss during menstruation, pregnancy and breastfeeding. It is worth noting H1-1 maintained consistently normal SF and TS during nearly three years of follow-up. This suggest that beyond gender and age, other factors such as environmental influences, lifestyle choice (insufficient dietary heme iron content), or genetic factors (potential modifier genes) may affect disease penetrance [4, 5].

Among the p.Y333H mutation heterozygotes in Table 1, all patients who progressed to organ damage showed SF levels > 1000 µg/L and TS > 90%. This implies that high SF levels correlate with the severity of iron deposition in tissues and organs, particularly SF levels > 1000 µg/L. This observation is consistent with relevant guidelines, indicating that patients with SF > 1000 µg/L or elevated aminotransferases have an increased risk of developing cirrhosis [2, 18]. Some studies have found that high SF levels are independently associated with an increased prevalence of diabetes in HC patients [19]. Therefore, for patients with SF levels > 1000 µg/L, it is necessary to prioritize screening for hemochromatosis and conduct a comprehensive evaluation for potential organ damage.

In vitro functional studies have demonstrated that the p.Y333H-mutated FPN can be localized on the cell membrane and retain its capacity to export iron outward. After interacting with hepcidin, the mutated FPN remained on the cell membrane and maintained iron export [12]. These findings suggest that the p.Y333H mutation alters the hepcidin receptor function of FPN, resulting in resistance to hepcidin-mediated internalization. Consequently, it reveals that the p.Y333H mutation in the *SLC40A1* gene is a gain-of-function mutation, aligning with the biochemical and clinical manifestations of HC patients with this genotype.

The correlation between the genotype and phenotype of *SLC40A1* gene variants has been further elucidated with the progression in FPN structure and function research. On the secondary structure, FPN consists of two 6-transmembrane-helix bundles, named as N-lobe and C-lobe, forming a central cavity for iron transport [20, 21]. Further research indicates that residues F324, C326, Y333, D504 and H507 in the C-lobe are essential binding and docking sites for hepcidin [22, 23]. Mutations at these sites lead to strong resistance to hepcidin. Three-dimensional (3D) structural studies propose that the Y333 residue fits into the hydrophobic cavity of hepcidin, forming hydrogen bonds with the backbone carbonyl of hepcidin M21 residue [24]. According to 3D models of FPN, mutations of Y333A, Y333H and Y333F that maintain normal binding to hepcidin may interfere with the conformation of FPN and impede its ubiquitination, resulting in the inability of FPN internalization [6, 12].

In conclusion, we report the largest pedigree with heterozygous *SLC40A1* p.Y333H mutation in the Chinese population to date. We have summarized the phenotypic manifestations and genetic pattern of *SLC40A1*-related HC with this mutation. Heterozygotes of this mutation exhibit a seemingly more complete penetrance in males over 30 years old, suggesting that genetic screening should be performed on the first-degree relatives of mutation heterozygotes, especially males in this age group, as they may already be in the clinical onset stage.

## Data Availability

The sequence data generated during the current study have been deposited in the Genome Sequence Archive for Human repository under accession code HRA006434. The data are available from the following link: https://bigd.big.ac.cn/gsa-human/browse/HRA006434.

## Abbreviations

HC: Haemochromatosis
FPN: Ferroportin
SF: Serum ferritin
TS: Transferrin saturation
MRI: Magnetic resonance imaging
3D: Three-dimensional
